# Toxicity in Peripheral Nerves: An Overview

**DOI:** 10.3390/toxics9090218

**Published:** 2021-09-11

**Authors:** Wolfgang Grisold, Valentina Alda Carozzi

**Affiliations:** 1 Ludwig Boltzmann Institute for Experimental und Clinical Traumatology, Donaueschingenstraße 13, A-1200 Wien, Austria; grisoldw@gmail.com; 2 Experimental Neurology Unit, School of Medicine and Surgery, University of Milan Bicocca, Building U8, Room 1027, Via Cadore 48, 20900 Monza, Italy

**Keywords:** toxic agents, peripheral neuropathy, peripheral nerve, dorsal root ganglia, pathogenetic mechanisms

## Abstract

Introduction to a collection. This article is intended to introduce a collection of papers on toxic neuropathies. Toxic neuropathies can be caused by a variety of substances and by different mechanisms. Toxic agents are numerous and can be distinguished between drugs, recreational agents, heavy metals, industrial agents, pesticides, warfare agents, biologic substances and venoms. Toxic agents reach the nervous system by ingestion, transcutaneously, via the mucous membranes, parenterally and by aerosols. The most frequent types are cumulative toxicities. Other types are acute or delayed toxicities. Pathogenetic mechanisms range from a specific toxic substance profile causing axonal or demyelinating lesions, towards ion channel interferences, immune-mediated mechanisms and a number of different molecular pathways. In addition, demyelination, focal lesions and small fiber damage may occur. Clinically, neurotoxicity presents most frequently as axonal symmetric neuropathies. In this work, we present a panoramic view of toxic neuropathy, in terms of symptoms, causes, mechanisms and classification.

## 1. Introduction

Peripheral neuropathy is a significant clinical condition that affects 15% of the population over 40 years old in the United States. Peripheral neuropathy can be caused by hereditary or acquired conditions. This paper aims to provide a general description of the sources of toxicity in the peripheral nervous system (PNS), the clinical features of toxic neuropathy (TN) based on the BH criteria as well as the mechanisms, time course, remission, progression and severity of the intoxication.

TNs are produced by exogenous neurotoxic substances of different origin, as chemical and organic substances, and represent an important cause of acquired neuropathy.

TNs can be environmental, occupational, recreational or iatrogenic, among others. The incidence can be described based on a geographical and economic point of view: in developing countries, occupational and environmental causes, including the exposure to heavy metals, arsenic, and organophosphorus compounds, are prevalent. In high-income countries, the most common cause of TNs are alcohol-induced neuropathies and neuropathies related to drug toxicity, such as tuberculostatic, anti-arhythmic and anti-cancer drugs, in particular deriving from chemotherapy drugs, such as platinum drugs (cisplatin), taxanes (paclitaxel), vinka-alkaloids (vincristine) and proteasome inhibitors (bortezomib). Drugs used in medicine (see Table 1) may have toxic effects on the PNS and the drugs’ effectiveness has to be outweighed against neurotoxicity. The neurotoxicity of commonly used drugs and medications can be a dose-limiting factor.

TNs can be caused not only by a variety of substances but also by different mechanisms. The most frequent types are cumulative toxicities, while acute toxicities and delayed toxicities can also occur. The mechanisms range from a specific substance profile, which induces toxicity by accumulation or other mechanisms, towards acting on ion channels or other cellular pathways or even inducing immune reactions of different types.

The causes of TNs can be classified into various groups, including drugs, heavy metals, industrial agents, recreational agents, biological agents, pesticides, warfare agents, venoms and others (see Table 2). Usually the toxic substance reaches the PNS that is not protected by the blood–brain barrier parenterally, by ingestion or rarely through aerosols. Some substances can reach the nervous system by retrograde transport along axons. In addition, local mechanisms such as penetration through the skin, local diffusion, limb perfusion and dissemination in cavities have to be considered.

Clinically, the most commonly observed patterns are symmetric length-dependent neuropathies in various presentations, many of them due to axonal damage, although myelin damage has also been described. Little is known about the individual damage of cutaneous receptors and isolated small fiber damage.

The identification of a neurotoxic drug can be straightforward or also difficult and time consuming in some cases (as an example, the research on the Spanish oil syndrome). As a rule, the Bradford Hill (BH) criteria should be applied for the identification of possible neurotoxicity [1,2] (see below).

## 2. Sources of Entry into the Body

Toxins can enter the body in various ways. Most commonly by ingestion, with drinks, food and drugs. Parenteral ingestions are common for drugs. In addition, gases and aerosols can be toxic. There are also local toxicities: on the skin, via high-pressure devices and in individual compartments of the body, such as cavities (e.g., peritoneum), or in isolated parts of the body as in the case of limb perfusions. See Table 3 for a detailed list and examples of toxin entry sources.

## 3. Environmental Toxicity

In addition to direct toxic effects of substances, new types of toxicities also appeared which are related to the concentration of toxic substances in wastewater, well water and drinking water. As an example, traces of cancer drugs can also be found in small concentrations in drinking water. In particular, the increasing number of cancer outpatients (receiving chemotherapy) increases the contamination, as urine and waste enter the public system. Although not neurotoxic in these concentrations, toxic effects on lactating women and child development, as well as possible cumulative effects, cannot be excluded [4].

## 4. Clinical Features of TNs

Most of the TNs are length-dependent symmetric distal axonal neuropathies, generally with a slow and insidious progression. Focal toxicities are much rarer. The most common presentation of TNs involves the largest diameter/longest axons producing axonal degeneration associated with numbness, paraesthesia, or weakness in a stocking–glove distribution. In some conditions autonomic involvement also appears. Clinically, most TNs are typically sensory, but motor symptoms may also occur. Autonomic involvement is infrequent; pruritus rarely occurs as a neuropathic symptom [5,6], and infrequently erythromelalgia does too [7,8]. Neuropathic pain can often impair the symptoms.

The additional involvement of cranial nerve damage, as the optic [9,10] and cochlear nerves have has been reported [11]. Mononeuropathies typically occur after lead intoxication, and there may be an increased susceptibility for individual nerve lesions in generalized TNs. Less frequently, local toxicity, including paravasates during medical interventions [12], high-pressure injection of toxic substances in industrial procedures [13], limb perfusion for local tumors [14], contact toxins as biologic agents, such as jellyfish [15], have been described.

The nerve plexus is rarely affected by toxicity, except by local anaesthetics, either by direct toxicity, mechanical or vascular factors, such as vasoconstriction [16]. The autonomic system can be affected by several drugs, particularly vinca alkaloids and statins [17,18].

### 4.1. Acute Toxicity

Acute toxicity is rare and mediated by different pathways. An example is oxaliplatin toxicity (chemotherapy drug), which affects ion channels in the PNS and produces a rapid acute cold-dependent hyperalgesia shortly after infusion [19]. Another example is the acute and irreversible toxicity observed after intrathecal administration of the chemotherapy drug vincristine, which results in an irreversible and lethal myeloradiculopathy [20].

### 4.2. Cumulative Toxicity

Most conventional intoxications follow the chronic and cumulative pathway, suggesting a prolonged and more chronic course, which is defined by the total toxic dose of the drug/agent. The development of toxicity can be linear and dose-dependent or exponentially progressive. The Common Terminology Criteria for Adverse Events (CTCAE) [21] are used for the classification of drug toxicity. Examples are chemotherapeutic agents used in cancer chemotherapy, among others. Coasting is described in some agents, such as platinum drugs, where the toxic effects progress for a period, despite the cessation of drug treatment [22]. 

**Figure 1:**
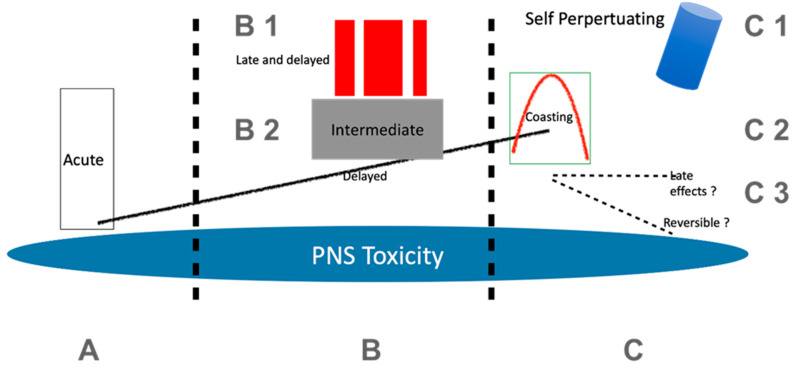
A schematic representation of the time course of toxic neuropathy caused by chemotherapy from exposure and onset of the toxicity on the PNS to the late effects and coasting phenomenon. Variable time course in patients receiving cancer treatment. The figure contains 3 critical time aspects (zones) for the development of chemotherapy-induced neurotoxicity (CIPN): (**A**): Acute toxicity is less frequent, and can be demonstrated in oxaliplatin toxicity. The acute toxic effects typically occur at the first intervention. (**B**): Cumulative toxicity, which usually follows the cumulation of the toxic agents’ chemotherapy cycle. Toxicity is usually incrementally increasing (symbolized by the black line). “Late and delayed” as well as intermediate toxicity stand for other patterns, such as immune-induced effects in ICI or intermediate effects caused by OPS. (**C**): Other late effects. After termination of chemotherapy, symptoms can progress for a variable time and then remit (“Coasting”). The extent of late effects in chemotherapy-induced neuropathies is an important question, as the number of cured patients and long-term survivors increases. In a few instances, such as TCE intoxication and also some OPS, progression after the termination of the exposure has also been described (self-perpetuation).

Figure 1 depicts a schematic representation of the time-course of toxic neuropathy caused by chemotherapy from exposure and onset of the toxicity on the PNS to the late-effects and coasting phenomenon.

### 4.3. Multiple Timely Presentations (Different Types of Toxicity of the Same Substance in Sequential Temporal Relationships)

Some substances, such as tri-chloro-ethilene (TCE), have a bi- or tri-modal course of action, which is an acute or subacute toxicity or an intermediate toxicity followed by a late toxicity, with different clinical patterns [23]. As an example, organophosphate intoxications can induce a cholinergic syndrome, such as the acute effect resulting in weakness of neck extensors, proximal muscles (including ventilatory muscles) and cranial nerves [24]. An intermediate effect as well as a late effect are represented in the organophosphate-induced delayed polyneuropathy, resulting in sensory symptoms, sensorimotor neuropathy and ataxia. Examples are several well-documented tri-ortho-cresyl phosphate intoxications [25].

### 4.4. Delayed Toxicity, Long-Term and Indirect Effects

Delayed toxicity has been increasingly reported in patients who have received conventional chemotherapies as long-term cancer survivors. Conventionally it is considered as a permanent and irreversible toxic effect. In CIPN, ongoing late immune mechanisms are discussed, which can kindle the persistence or even worsening of the neuropathy [26,27].

There are also indirect effects, based on immunological reactions, which appear with a variable delay. There are several other examples, where different types of mechanisms cause neuropathy: immune checkpoint inhibitors (ICI), where an autoimmune mechanism is activated, sulfonamides that rarely induce vasculitis-causing neuropathy [28], aerosols that can induce Guillain–Barrè syndrome (GBS) in swine abattoir workers [29] as well as the Spanish toxic oil syndrome, probably causing vasculitis [30], or the toxic gold treatments in rheumatology [31].

## 5. Pathogenesis and Prognosis

Pathologically, axons can be damaged, producing a primary axonopathy when the cytoarchitectural organization and the function of axons are directly damaged (as with some chemotherapeutic drugs). However, axonal damage can also derive secondarily after primary damage of peripheral neurons’ perykaria or after a primary demyelination (secondary axonopathy). This reflects the fact that large and long axons are ultimately affected whether or not they are the primary critical target. Demyelination can also occur due to Schwann cell impairment [32] as well as long-tract and dying-back phenomena. Multifocal and multiplex neuropathies caused by toxins are rare, as with those generated by vasculitis (e.g., caused by drugs) [33]. Most TNs are dose-dependent and reversible after a variable time after cessation of exposure. For the development of TNs, in addition to the substance and dose, several individual factors such as age, individual susceptibility, idiosyncrasy and concomitant diseases facilitating susceptibility and other conditions (e.g., malnutrition, diabetes and genetic neuropathies) have an influence on the severity of neuropathy. The role of the interaction of different conditions is not resolved: an example would be a patient with a diabetic neuropathy, receiving chemotherapy for cancer with drug A, followed by second-line chemotherapy with drugs B and C.

The information on the effects of combined therapies on the nervous system is limited [21,34]. Other examples are toxicities from venoms and tick bites, which often contain a combination of different substances.

## 6. Criteria Useful to Classify TNs: The “Bradford Hill Criteria”

The Bradford Hill (BH) criteria, known as Hill’s criteria for causation, were established in 1965 as a group of principles that can be useful in establishing epidemiologic evidence of a causal relationship between a presumed cause and an observed effect. They have been widely employed in medicine and research. The BH criteria have been successfully applied and are a useful instrument in the identification of TN. The list of the criteria is reported here from the original work of Bradford in 1965 [1]. Although very useful and robust, the BH criteria have been criticized and a modernization has been suggested [2,22].

The classical pathways to detect toxicity include a thorough history and electrophysiology. Imaging is rarely used. For the detection of toxic agents, blood (molecular blood biomarkers) and tissue investigations are used [35].

## 7. Targets and Mechanisms of Neurotoxicants

The mechanisms causing TNs are heterogenous and toxin-dependent. For many substances the mechanisms have been explored and often experimentally confirmed, primarily based on morphological, functional and molecular analysis of dorsal root ganglia (DRG) and peripheral nerves, identifying several determinants in the establishment of peripheral toxic damage (i.e., mitochondrial dysregulation, ROS generation, microtubule dysfunction, cytoskeletal alterations, ion channels and membrane transporter alterations). Here we consider the main targets of toxicants in the PNS, describing some of the main mechanisms involved in peripheral neurotoxicity and giving some examples.

### 7.1. The Nerve Axon as a Target of Neurotoxins

The physiological process of nerve impulse propagation requires a preserved structure of axons, in terms of anatomical organization and molecular as well as biochemical homeostasis. The alteration of the cytoarchitecture of axons and/or of their functionality is a cause of axonopathy. Primary axonopathy is the most common PNS damage during or after exposure to neurotoxins. Since the neuronal cell body is the center of synthesis of the neuron, where macromolecules, including neurotransmitters and organelles, such as mitochondria, are synthesized, the axonal transport is essential for distribution along axons. Alterations in the dynamics of alpha- and beta-tubulin assembly and disassembly along axons, which maintain the organization and function of microtubules, cause axonopathy. Some neurotoxicants, such as cancer chemotherapy drugs (anti-tubulins and vinca alkaloids) and certain environmental chemicals (n-Hexane, carbon disulphide and acrylamide) act through this mechanism, causing a primary axonopathy [36,37,38,39,40,41]. Other cytoskeletal filaments can be impaired, such as actin microfilaments and neurofilaments (neuronal intermediate filaments). At the electron microscopic level microtubular abnormalities can be observed, as can the formation of clear vacuoles, macrophage infiltration and Schwann cell alterations [42,43,44,45].

### 7.2. Schwann Cells and Myelin as Targets of Neurotoxicity

As previously reported, the physiological process of nerve impulse propagation along axons requires a preserved structure and organization. Schwann cells, being supportive, and trophic cells, for neuronal structural and functional maintenance, are key actors in nerve homeostasis. A variety of molecules has been implicated in the signaling between peripheral axons and Schwann cells (i.e., myelin-associated glycoprotein [46], low-affinity nerve growth factor receptor (p75) [47], insulin-like growth factor 1, transforming growth factor beta, growth factor neuregulin 1 (NRG1) and the erbB receptors [48,49]). Defects in these signaling pathways can be associated with defects in nerve functionality and impulse propagation. Myelinopathies primarily occur if myelin is primarily damaged, or secondarily following axonopathy.

An example of agents causing myelinopathies is hexachlorophene contained in detergents and soaps, which is able to enter by the skin, reaching the central nervous system (CNS) and PNS, especially in newborns where the blood–brain barrier is incompletely formed [50,51]. Another example is triethyltin as a contaminant in medication and industrial pollutant [52]. A myelin swelling is the typical morphological lesion with a splitting of intraperiod lines.

### 7.3. Peripheral Neurons and Satellite Glial Cells as Targets of Neurotoxicity

Peripheral neuronal perikarya (located in autonomic as well as spinal ganglia) can be affected by neurotoxicants, which produce injury and cell death. This condition is named “neuronopathy”. The damage can be primary or secondary to axonopathy. In the first case, the soma of neurons are the target site of neurotoxicants that affect the nuclear and cytoplasmic neuronal machinery (mitochondria, ribosomes, endoplasmic reticulum, detoxification mechanisms, etc.). Severe damage causes neuronal death, associated with an irreversible loss of nerve fibers. In the second case, in which neuronal damage is secondary to fiber damage, the retrograde signaling of injury from the axon results in the neuronal “chromatolysis response”, which consists of neuronal enlargement, rounding of the cytoplasmic membrane, eccentric displacement of the nucleus and loss of Nissl substance [53,54].

Apoptosis is a pathway of programmed cell death, which requires energy and specific protein regulation that control the process. As an example, platinum-based chemotherapeutic agents produce cell death in DRG neurons through apoptosis.

The presence of a high concentration of blood-fenestrated capillaries and the absence of the blood–brain barrier allow their preferential access to this region of the DRG. A different mechanism is the acute toxicity of oxaliplatin, which alters the kinetics of the neuron voltage-gated sodium ion channels [55,56,57,58]. This effect is thought to contribute to the acute clinical signs of cold paresthesia observed in patients after oxaliplatin infusion. The activation of transient receptor potential (TRP) channels (i.e., vanilloid type one, TRPV1) contributes to the development of neuropathic pain [59].

Beside neurons, glial satellite cells (SGC) can also be affected by neurotoxic substances as they are accessible as neurons in the PNS. These cells are in intimate relationships with neurons and have a trophic, important key role in supporting neuronal nutrition, function and survival. Generally, they seem to be less susceptible than neurons to damage. An increase in number (i.e., gliosis) was described after toxic damage with some chemotherapeutic agents (taxanes and oxaliplatin) in order to meet increased metabolic needs of neurons [60].

### 7.4. Other Mechanisms

Other mechanisms of neurotoxicity can involve the nodes of Ranvier, as is described for amiodarone, an anti-arrhythmic medication [61]. Immune-mediated mechanisms can cause a delayed toxicity by inducing an immune-mediated neuropathy (vasculitis, GBS, see 7.d), which can be considered to be an indirect mechanism. These and other examples of toxic agents, sites of action and mechanisms are reported in Table 4.

## 8. Conclusions

This special issue deals with TNs from several perspectives and attempts to introduce their complexity in terms of multiple causes, agents, symptoms, mechanisms, targets and prognosis.

For the clinician it is helpful to elucidate that TNs are not restricted to the often-assumed cumulative toxicity, but there are also other presentations, such as acute forms, multimodal presentations and late effects, in addition to increasingly indirect as well as remote effects.

The mechanisms are highly substance-dependent, but also vary considerably, and are often not only dose-, but also time-dependent. As new and emerging toxicities appear, delayed effects of therapy, often acting on an immune mechanism, have been cited. The capability of an agent to target the PNS belongs to its ability to cross the blood–nerve barrier, gaining access to nerve endings, nerves and ganglia. Once these targets are reached, the information can rapidly be conducted throughout the PNS as well as into the CNS, propagating pathologic information.

Mechanisms of diseases are manifold, even if symptoms can be similar. In CIPN, for instance, different neurotoxic drugs acting on several different cellular pathways (e.g., microtubule alterations, DNA alkylation and proteasome inhibition) can generate similar pathologic phenotypes in patients and animal models.

One possible new aspect is the toxicity of the environment by drinking well and surface water, which gains importance as cancer therapies are usually performed in outpatients and excretions are added to wastewater. These studies are also extended towards healthcare professionals and their exposure.

The identification of toxicity needs a careful process that considers and excludes chance associations. The identification can be substantiated by using the BH criteria. The key to therapy is the identification of the toxic substance, according to the BH, and active interventions such as the removal of the toxic agents, rarely application of antidotes and managing delayed effects. CIPN is a good example of neurotoxicity where survival of cancer patients has been significantly increased, albeit in addition to the classical toxicity, increasingly late effects have appeared, which pose new issues towards the research on TNs.

## Figures and Tables

**Table 1:** List of the most common drugs that can cause toxic neuropathies. The list is not complete and does not distinguish between the different mechanisms and phenotypes. The order is alphabetical.

Anti-Microbials	Anti-Cancer Drugs	Cardiovascular	Psychiatric/Central Nervous System Disorders	Vitamins	Others
Chloroquine	Brentuximab vedotin	Amiodarone	Chlorprothixene	Vitamin B6 overdose	Allopurinol
Chloramphenicol	Epothilones	Perhexilene	Glutethimide	Vitamin B12 deficiency	Colchicin
Dapsone	Platinum drugs (cisplatin and oxaliplatin)	Propafenone	Phenelzine		Cyclosporin A
Ethambutol	Proteasome inhibitors (bortezomib)	Statins	Phenytoin		Dichloroacetate
Fluoroquinolone	Taxanes (paclitaxel and docetaxel)				Disulfiram
Linezolid	Trastuzumab emtansine				Etanercept
Metronidazol	Vinca alkaloids (vincristine and vinblastine)				Gold
Nitrofurantoin					Hydralazine
Nucleoside analogues					Infliximab
Sulfasalazin					Interferon alpha
Tuberculostatics					Leflunomid
					D-penicillamine
					Tacrolimus

**Table 2:** List of the most common toxic substance groups that can produce toxic neuropathies. Alphabetical order.

Substance Groups	Examples
Anti-freeze substances	Diethylen glycole and methylbromide
Biological agents and venoms	Brevetoxin, ciguatera, domoic acid, lara toxin, saxitoxin, snake and spider venoms as well as tetrodoxin
Drugs, medicines and anesthesiology drugs	See dedicated table (Table 2), nitrous oxide Local toxicity (various agents)
Environment, water sources and wastewater	Wells: As, dioxin and Hg Chemotherapy excretions in wastewater
Food and diet	Examples: Spanish oil syndrome Fish poisoning
Industrial agents	Acrylamide, hexacarbone and solvents
Heavy metals	As, Hg, Pb, Th and Zn
Pesticide and herbicides	Dioxin, organophosphate and vacor
Plants	Example: sea buckthorn berry
“Recreational drugs”	Alcohol, methanol, (glue) “sniffing”

**Table 3:** List of the most common sources and ways of entry of toxic substances able to produce toxic neuropathies. (#) The influence of environmental toxins has been examined in hospital personnel, as well as in large studies in well and wastewater [3] for cancer drugs. (+) Grouting is a high-pressure technique used in buildings.

Source/Entry	Site of Entry	Examples
Aerosols	Air tract	Aerosols, glue, NO and solvents
Ingestion	Mouth and intestine	Drugs, Food, Fluids (alcohol)
Local	Skin Paravasate Cavities—local toxicity and dissemination (e.g., intraperitoneal) Perfusion (e.g., limbs)	Local High-pressure device Grouting (+) (e.g., acrylamide) Anti-cancer drugs IT or IP chemotherapy Local tumor perfusion
Parenteral	Bloodstream	Medical treatment, drugs, IV and IT
Environmental (#)	Air Contamination Fumes Well water	Different dimensions of concentrations: see discussion

**Table 4:** Sites and mechanisms for peripheral neurotoxicity. Axonopathies and myelinopathies are the most frequently observed mechanisms.

Target Site	Mechanisms	Toxic Substances	Examples
Axon	Affected transport along axons Affected microtubule assembly Defects in neurofilament and actin microfilaments	Chemotherapeutic drugs Environmental chemicals	Adriamicine Anti-tubulin Bortezomib Epothilones Vinca alkaloids n-Hexane Carbon disulphide Acrylamide
Myelin and Schwann cells	Defects in key molecules for axon–Schwann cell signaling	Chemotherapy drugs Other drugs Adjuvants in soaps Contaminants in medication	Bortezomib Suramin Adalimumab Amiodorone Etanercept Infliximab Hexaclorophene Perhexiline Triethyltin
DRG	Organelle damage (mitochondria, ER, proteasome, etc.) Nuclear and mitochondrial DNA damage Defects in ion channels and receptors Defects in neuron–SGC signaling	Chemotherapeutic drugs Other drugs Venoms Vitamin excess	Bortezomib Platinum compounds Thalidomide Nitrofurantoin Isoniazid Mercury Pyridoxamine
Immune-mediated	Secondary induction of an immune response	Chemotherapeutic drugs Environmental substances	Immune checkpoint inhibitors

## Data Availability

Not applicable.

## References

[1] Hill A.B. (1965). The Environment and Disease: Association or Causation?. Proc. R. Soc. Med..

[2] Hill A.B. (2015). The environment and disease: Association or causation? 1965. J. R. Soc. Med..

[3] Franquet-Griell H., Gómez-Canela C., Ventura F., Lacorte S. (2015). Predicting concentrations of cytostatic drugs in sewage effluents and surface waters of Catalonia (NE Spain). Environ. Res..

[4] Moretti M., Bonfiglioli R., Feretti D., Pavanello S., Mussi F., Grollino M.G., Villarini M., Barbieri A., Ceretti E., Carrieri M. (2011). A study protocol for the evaluation of occupational mutagenic/carcinogenic risks in subjects exposed to antineoplastic drugs: A multicentric project. BMC Public Health.

[5] Hachisuka J., Chiang M.C., Ross S.E. (2018). Itch and neuropathic itch. Pain.

[6] Oaklander A.L. (2012). Common neuropathic itch syndromes. Acta Derm. Venereol..

[7] Saviuc P.F., Danel V.C., Moreau P.A., Guez D.R., Claustre A.M., Carpentier P.H., Mallaret M.P., Ducluzeau R. (2001). Erythromelalgia and mushroom poisoning. J. Toxicol. Clin. Toxicol..

[8] Cimolai N., Cimolai T. (2009). Erythromelalgia accompanying rosuvastatin-associated myopathy. J. Dermatol. Case Rep..

[9] Grzybowski A., Zulsdorff M., Wilhelm H., Tonagel F. (2015). Toxic optic neuropathies: An updated review. Acta Ophthalmol..

[10] Wasinska-Borowiec W., Aghdam K.A., Saari J.M., Grzybowski A. (2017). An Updated Review on the Most Common Agents Causing Toxic Optic Neuropathies. Curr. Pharm. Des..

[11] Lindhard Madsen M., Du H., Ejskjær N., Jensen P., Madsen J., Dybkær K. (2019). Aspects of vincristine-induced neuropathy in hematologic malignancies: A systematic review. Cancer Chemother. Pharmacol..

[12] Fehm T., Marme A., Lipp H.P., Schumacher K. (2008). Paravasation von Zytostatika. Der Gynäkologe.

[13] Emre A.U. (2009). Median Nerve Injury Due to High-Pressure Water Jet Injection: A Case Report and Review of Literature. Eur. J. Trauma Emerg. Surg..

[14] Busse O., Aigner K., Wilimzig H. (1983). Peripheral nerve damage following isolated extremity perfusion with cis-platinum. Recent Results Cancer Res..

[15] Peel N., Kandler R. (1990). Localized neuropathy following jellyfish sting. Postgrad. Med J..

[16] Hebl J.R., Horlocker T.T., Pritchard D.J. (2001). Diffuse Brachial Plexopathy after Interscalene Blockade in a Patient Receiving Cisplatin Chemotherapy: The Pharmacologic Double Crush Syndrome. Anesth. Analg..

[17] Novak P., Pimentel D.A., Sundar B., Moonis M., Qin L., Novak V. (2015). Association of Statins with Sensory and Autonomic Ganglionopathy. Front. Aging Neurosci..

[18] Giannoccaro M.P. (2011). Somatic and autonomic small fiber neuropathy induced by bortezomib therapy: An immunofluorescence study. Neurol. Sci..

[19] Gebremedhn E.G., Shortland P.J., Mahns D.A. (2018). The incidence of acute oxaliplatin-induced neuropathy and its impact on treatment in the first cycle: A systematic review. BMC Cancer.

[20] Alcaraz A., Rey C., Concha A., Medina A. (2002). Intrathecal vincristine: Fatal myeloencephalopathy despite cerebrospinal fluid perfusion. J. Toxicol. Clin. Toxicol..

[21] Otsuka R., Iwasa S., Yanai T., Hirano H., Shoji H., Honma Y., Okita N., Takashima A., Kato K., Hashimoto H. (2020). Impact of peripheral neuropathy induced by platinum in first-line chemotherapy on second-line chemotherapy with paclitaxel for advanced gastric cancer. Int. J Clin. Oncol..

[22] Cox L.A. (2018). Modernizing the Bradford Hill criteria for assessing causal relationships in observational data. Crit. Rev. Toxicol..

[23] Abdollahi M., Karami-Mohajeri S. (2012). A comprehensive review on experimental and clinical findings in intermediate syndrome caused by organophosphate poisoning. Toxicol. Appl. Pharmacol..

[24] Haliga R.E., Morarasu B.B., Ursaru M., Irimioaia V., Sorodoc L. (2018). New insights into the organophosphate-induced intermediate syndrome. Arh. Hig. Rada. Toksikol..

[25] Vasconcellos L.F., Leite A.C., Nascimento O.J. (2002). Organophosphate-induced delayed neuropathy: Case report. Arq. Neuropsiquiatr..

[26] Agalave N.M., Mody P.H., Szabo-Pardi T.A., Jeong H.S., Burton M.D. (2021). Neuroimmune Consequences of eIF4E Phosphorylation on Chemotherapy-Induced Peripheral Neuropathy. Front. Immunol..

[27] Fumagalli G., Monza L., Cavaletti G., Rigolio R., Meregalli C. (2021). Neuroinflammatory Process Involved in Different Preclinical Models of Chemotherapy-Induced Peripheral Neuropathy. Front. Immunol..

[28] Lehr D. (1972). Sulfonamide vasculitis. J. Clin. Pharmacol. New Drugs..

[29] Adjemian J., Howell J., Holzbauer S., Harris J., Recuenco S., McQuiston J., Chester T., Lynfield R., Devries A., Belay E. (2009). A clustering of immune-mediated polyradiculoneuropathy among swine abattoir workers exposed to aerosolized porcine brains, Indiana, United States. Int. J. Occup. Environ. Health.

[30] Gelpi E., Posada de la Paz M., Terracini B., Abaitua I., Gómez de la Cámara A., Kilbourne E.M., Lahoz C., Nemery B., Philen R.M., Soldevilla L. (2002). The Spanish toxic oil syndrome 20 years after its onset: A multidisciplinary review of scientific knowledge. Environ. Health Perspect..

[31] Grisold W., Mamoli B. (1984). The syndrome of continuous muscle fibre activity following gold therapy. J. Neurol..

[32] Jortner B.S. (2000). Mechanisms of toxic injury in the peripheral nervous system: Neuropathologic considerations. Toxicol. Pathol..

[33] Kist A.M., Sagafos D., Rush A.M., Neacsu C., Eberhardt E., Schmidt R., Lunden L.K., Ørstavik K., Kaluza L., Meents J. (2016). SCN10A Mutation in a Patient with Erythromelalgia Enhances C-Fiber Activity Dependent Slowing. PLoS ONE.

[34] Carozzi V., Chiorazzi A., Canta A., Oggioni N., Gilardini A., Rodriguez-Menendez V., Avezza F., Crippa L., Ceresa C., Nicolini G. (2009). Effect of the chronic combined administration of cisplatin and paclitaxel in a rat model of peripheral neurotoxicity. Eur. J. Cancer.

[35] Meregalli C., Bonomo R., Cavaletti G., Carozzi V.A. (2021). Blood molecular biomarkers for chemotherapy-induced peripheral neuropathy: From preclinical models to clinical practice. Neurosci. Lett..

[36] Benbow S.J., Cook B.M., Reifert J., Wozniak K.M., Slusher B.S., Littlefield B.A., Wilson L., Jordan M.A., Feinstein S.C. (2016). Effects of Paclitaxel and Eribulin in Mouse Sciatic Nerve: A Microtubule-Based Rationale for the Differential Induction of Chemotherapy-Induced Peripheral Neuropathy. Neurotox. Res..

[37] Smith J.A., Slusher B.S., Wozniak K.M., Farah M.H., Smiyun G., Wilson L., Feinstein S., Jordan M.A. (2016). Structural Basis for Induction of Peripheral Neuropathy by Microtubule-Targeting Cancer Drugs. Cancer Res..

[38] Herskowitz A., Ishii N., Schaumburg H. (1971). n-Hexane neuropathy: A syndrome occurring as a result of industrial exposure. N. Engl. J. Med..

[39] Rizzuto N., De Grandis D., Di Trapani G., Pasinato E. (1980). n-Hexane polyneuropathy: An occupational disease of shoemakers. Eur. Neurol..

[40] Chang Y.C. (1990). Patients with n-hexane induced polyneuropathy: A clinical follow up. Br. J. Ind. Med..

[41] Huang C.C. (2008). Polyneuropathy induced by n-hexane intoxication in Taiwan. Acta Neurol. Taiwanica.

[42] De Waegh S.M., Lee V.M., Brady S.T. (1992). Local modulation of neurofilament phosphorylation, axonal caliber, and slow axonal transport by myelinating Schwann cells. Cell.

[43] Mata M., Kupina N., Fink D.J. (1992). Phosphorylation-dependent neurofilament epitopes are reduced at the node of Ranvier. J. Neurocytol..

[44] Hsieh S.T., Kidd G.J., Crawford T.O., Xu Z., Lin W.M., Trapp B.D., Cleveland D.W., Griffin J.W. (1994). Regional modulation of neurofilament organization by myelination in normal axons. J. Neurosci..

[45] Shemesh O.A., Spira M.E. (2010). Paclitaxel induces axonal microtubules polareconfiguration and impaired organelle transport: Implications for the pathogenesis of paclitaxel-induced polyneuropathy. Acta Neuropathol..

[46] Yin X., Crawford T.O., Griffin J.W., Tu P.H., Lee V.M., Li C., Roder J., Trapp B.D. (1998). Myelin-associated glycoprotein is a myelin signal that modulates the caliber of myelinated axons. J. Neurosci..

[47] Cosgaya J.M., Chan J.R., Shooter E.M. (2002). The neurotrophin receptor p75NTR as a positive modulator of myelination. Science.

[48] Syroid D.E., Maycox P.R., Burrola P. (1996). Cell death in the Schwann cell lineage and its regulation by neuregulin. Proc. Natl. Acad. Sci. USA.

[49] Guenard V., Gwynn L.A., Wood P.M. (1995). Transforming growth factor-beta blocks myelination but not ensheathment of axons by Schwann cells in vitro. J. Neurosci..

[50] Towfighi J., Gonatas N.K., McCree L. (1974). Hexachlorophene-induced changes in central and peripheral myelinated axons of developing and adult rats. Lab. Investig..

[51] Tripier M.F., Berard M., Toga M., Martin-Bouyer G., Le Breton R., Garat J. (1981). Hexachlorophene and the central nervous system. Toxic effects in mice and baboons. Acta Neuropathol..

[52] Graham D.I., de Jesus P.V., Pleasure D.E., Gonatas N.K. (1976). Triethyltin sulfateinduced neuropathy in rats. Electrophysiologic, morphologic, and biochemical studies. Arch. Neurol..

[53] Hanz S., Fainzilber M. (2006). Retrograde signaling in injured nerve–the axon reaction revisited. J. Neurochem..

[54] Scheib J., Ho¨ke A. (2013). Advances in peripheral nerve regeneration. Nat. Rev. Neurol..

[55] Adelsberger H., Quasthoff S., Grosskreutz J., Lepier A., Eckel F., Lersch C. (2000). The chemotherapeutic oxaliplatin alters voltage-gated Na(þ) channel kinetics on rat sensory neurons. Eur. J. Pharmacol..

[56] Park S.B., Lin C.S.Y., Krishnan A.V., Goldstein D., Friedlander M.L., Kiernan M.C. (2011). Dose effects of oxaliplatin on persistent and transient Naþ conductances and the development of neurotoxicity. PLoS ONE.

[57] Wu S.N., Chen B.S., Wu Y.H., Peng H., Chen L.T. (2009). The mechanism of the actions of oxaliplatin on ion currents and action potentials in differentiated NG108-15 neuronal cells. Neurotoxicology.

[58] Webster R.G., Brain K.L., Wilson R.H., Grem J.L., Vincent A. (2005). Oxaliplatin induces hyperexcitability at motor and autonomic neuromuscular junctions through effects on voltage-gated sodium channels. Br. J. Pharmacol..

[59] Quartu M., Carozzi V.A., Dorsey S.G., Serra M.P., Poddighe L., Picci C., Boi M., Melis T., Del Fiacco M., Meregalli C. (2014). Bortezomib treatment produces nocifensive behavior and changes in the expression of TRPV1, CGRP, and substance P in the rat DRG, spinal cord, and sciatic nerve. Biomed Res. Int..

[60] Warwick R.A., Hanani M. (2013). The contribution of satellite glial cells to chemotherapy-induced neuropathic pain. Eur. J. Pain.

[61] Ali M.U., Fitzpatrick-Lewis D., Kenny M., Raina P., Atkins D.L., Soar J., Nolan J., Ristagno G., Sherifali D. (2018). Effectiveness of antiarrhythmic drugs for shockable cardiac arrest: A systematic review. Resuscitation.

